# A formal model for analyzing drug combination effects and its application in TNF-α-induced NFκB pathway

**DOI:** 10.1186/1752-0509-4-50

**Published:** 2010-04-25

**Authors:** Han Yan, Bo Zhang, Shao Li, Qianchuan Zhao

**Affiliations:** 1 Department of Automation and TNList, Tsinghua University, Beijing, 100084, China

## Abstract

**Background:**

Drug combination therapy is commonly used in clinical practice. Many methods including Bliss independence method have been proposed for drug combination design based on simulations models or experiments. Although Bliss independence method can help to solve the drug combination design problem when there are only a small number of combinations, as the number of combinations increases, it may not be scalable. Exploration of system structure becomes important to reduce the complexity of the design problem.

**Results:**

In this paper, we deduced a mathematical model which can simplify the serial structure and parallel structure of biological pathway for synergy evaluation of drug combinations. We demonstrated in steady state the sign of the synergism assessment factor derivative of the original system can be predicted by the sign of its simplified system. In addition, we analyzed the influence of feedback structure on survival ratio of the serial structure. We provided a sufficient condition under which the combination effect could be maintained. Furthermore, we applied our method to find three synergistic drug combinations on tumor necrosis factor α-induced NFκB pathway and subsequently verified by the cell experiment.

**Conclusions:**

We identified several structural properties underlying the Bliss independence criterion, and developed a systematic simplification framework for drug combiation desgin by combining simulation and system reaction network topology analysis. We hope that this work can provide insights to tackle the challenging problem of assessment of combinational drug therapy effect in a large scale signaling pathway. And hopefully in the future our method could be expanded to more general criteria.

## Background

Drug combination therapy is commonly used in clinical practice [1-3]. For example, herbal remedies in traditional Chinese medicine are believed to have synergism effect [4,5]. How to define the drug synergism has been a long-standing controversy amongst pharmacologists, toxicologists and biologists [6,7]. Among existing methods, under the assumption that two drugs acting by independent mechanisms, Bliss independence model is used to define combined effect of two drugs [8]; given that two similar drugs competitively acting on a target, Loewe additivity model is used to predict the combined effect of two drugs [9]. Chou-Talalay further proposed the Combination Index (CI) theorem, serving as a general expression and quantification of drug interaction based on the mass-action law in biophysics and biochemistry [10-12]. These models are widely used in *in-vitro *and *in-silico *experiments for drug targets design and dose-response relationship analysis to instruct the selection of drugs and design of combination scheme [13-15]. Although improvements in the application scope and sensitivity of synergy evaluation techniques allow a greater exploitation of drug combination studies, it is unlikely that experimental techniques will be sufficient to completely screen the vast space of drug combinations in a cost-effective and timely manner. Hence, finding a way to delimit this space and obtain a manageable set of synergistic combinations is still an ongoing challenge.

To meet this challenge, we present a new method developed from the original Bliss independence criterion to analyze the relationship between structures and effects for combinational drug targets design from a mathematical aspect. Since Bliss model is relatively simple and still widely used by some researchers recently [16-18], we start our studies from this simple model and operationally provide a combination effect assessment index inspired by the Combination Index theorem [10-12]. With a foundational property of this index, the structure information could be used to help the analysis of drug combinational effects and design of combiantional drug targets. Under this frame, we study two classic structures (serial and parallel structures) in biological signaling systems and propose simplification rules which are helpful for analyzing drug combination effects on the original system. Furthermore, analysis of the feedback structures, which is also very common in signaling pathways, is conducted as an expansion to an original structures without feedback. The usefulness of all the results is demonstrated by numerical experiments.

As a concrete example, we applied our method to an inflammatory angiogenesis-related pathway, the tumor necrosis factor α (TNF-α)-induced NFκB pathway. The comprehensive research of this pathway has accumulated abundant exprimental data. This allows us to construct a TNF-α-induced NFκB pathway model. Here, we further extended previous model to endothelial cells to construct a more accurate model for drug efficacy prediction. With this new NFκB model in hand, we simualted the combined effects of three important inhibitors, namely Aldehyde, Geldanamycin and PS-1145, in NFκB pathway. The simulation results suggested that three inhibitor combinations yeilded significant synergism and were validated the simulated results by cell experiments.

## Methods

### Original Bliss Independence Criterion

Bliss independence [8] or fractional product method [19] is the index for calculating the expected dose-response relationship for drug combination therapy as compared to mono-therapy. It assumes that the two inhibitors act via independent mechanisms. Then drug combination can be represented as the union of two probabilistically independent events. And this criterion is identical to the mutually non-exclusive case [20]. The combined effect of two inhibitors (*F*_*UA*_) is computed as the product of individual effects of the two inhibitors, *F*_*UA*1 _and *F*_*UA*2_.(1)

where *F*_*UA *_is the remaining enzyme activity (fractional unaffected).

Based on the definition, *F*_*UA *_is the expected combined effect. If the actual combined effect of the two inhibitors is equal to *F*_*UA*_, it is additive effect case and there is no interaction between the two inhibitors. If the actual combined effect is lower than *F*_*UA*_, it is called antagonism. If the actual combined effect is higher than *F*_*UA*_, it is called synergism which leads many possible favorable outcomes like increasing or maintaining drug efficacy as decreasing dosage and provides fundations to the combination therapy [20].

### Survival ratio

We use survival ratio as representation of the effect (fractional unaffected) and define it as the ratio of component concentrations before and after intervention:(2)

where *a*, *b *are parameters that could be affected. Often they have relationship with the inhibitor doses. *a*_0 _and *b*_0 _represent the normal values, which are the values before being inhibited; *a *and *b *represent the values after being inhibited. The output of a system is usually defined as the concentration of some components.

Inspired by the Combination Index theorem offered by Chou-Talalay [10,11], here we introduce an operational concept, "Synergism Assessment Factor", for addressing the interaction of drug combination. Then the Bliss independence criterion could be rewritten as:(3)

Where *r*(*a, b*) is the actual combined effect and *r*(*a*_0_, *b*)·*r*(*a, b*_0_) is the expect combined effect calculated by the product of individual effects. *S *denotes Synergism Assessment Factor. Eq. 3 is identical to the fraction product equation of Webb [19] and the mutually non-exclusive case in [21]. Compared with the critical point (CI = 1) of Chou-Talalay's Combination Index, we used *S *= 0 as the critical point to determine whether there is sysnergistic effect. Under the first order mutually non-exclusive case, using Eq.3 will get the same conclusion on combination effect as using Combination Index.

Survival ratios of individual invention and combined invention can be measured through *in-vitro *or *in-silico *experiments, so it is convenient to verify whether synergism is generated under specific drug combination with this criterion [22]. Therefore, it is widely used in combination therapy design [16-18]. However, it is hard to predict the combined effect of two inhibitors without experiments according to this model itself. In order to predict the proper dose range to generate synergism, we have to gain the dose-response relationship. The dose-response relationship could be get through series of *in-vitro *experiments costly under different doses. Sometimes the dose-response relationship is assumed to have some special form like Hill equation to reduce the experiment costs [22]. It is feasible when possible targets number is small. As the targets number increases, it will face the combinatorial explosion to choose targets and proper doses, and the experiments cost also increases. New method to narrow down the possibilities in searching targets and doses generating synergism by experiments needs to be developed.

### Extended Bliss Independence Criterion

Here, we extend Bliss independence criterion with the sensitivity information of Synergism Assessment Factor. The system with some special structures could be simplified for synergism-generating targets with doses based on this criterion. We define:(4)

where, *r*(*a, b*) is the survival ratio of the system; *a *and *b *are parameters affected by inhibitor (ususally the reaction velocity constants [13]), *a*_0 _and *b*_0 _are separately the value of unaffected. Here *DS *denotes Synergism Assessment Factor Derivative. Actually *DS *is the second-order patial derivative of *S*. Our criterion is stated as(5)

This criterion is based on the following observations. The connection between *DS *and *S *is:(6)

It is easy to see that if *DS *< 0 (for the interested drug dose ranges), then *S *is guaranteed to be smaller than 0. It means that synergism is generated over the parameter ranges (*a*_0_, *a*), (*b*_0_, *b*). Similarly, the condition to generate antagonism is also intuitive.

This extended Bliss independence criterion could be seen as the derivative form of the original Bliss independence criterion.

### Fundamental property of synergism assessment factor derivative

The extended Bliss independence criterion introduced in Eq. 5 enables us to find special structures of systems that simplification is possible for synergism-generating targets with doses. Here we provide a basic property of the synergism assessment factor derivative *DS*. It is the foundation for our main results.

If the inhibitors individually affect some intermediate processes, then the inhibition on these processes could be taken as directly on the products of these processes. That is to say, *x *= Φ (*a*) is the product of the process where parameter *a *is affected; *y *= Ψ (*b*) is the product of the process where parameter *b *is affected. Then the inhibition effects to the output of system on *a *and *b *could be taken as on *x *and *y*. The analysis of combination effects on the original system could then be limited on a simplified system with *x *and *y *as parameters.

#### Lemma 1

*a *and *b *are system parameters that will be affected by inhibitors. *a *is of processes that will produce product *x *(*x *= Φ (*a*)), and *b *is of processes that will produce product *y *(*y *= Ψ (*b*)). Then the synergism assessment factor derivative *DS *of the original system satisfies

Where , *x *= Φ (*a*), *y *= Ψ (*b*), *x*_0 _= Φ (*a*_0_), *y*_0 _= Ψ (*b*_0_). Details of proof are given in Additional file 1.

*DS *is the synergism assessment factor derivative of the original system while *DS*' could be seen as that of a simplified system. Usually the signs of derivatives  and  are easy to know, then the sign of *DS *could be decided on the sign of *DS*'; and we only need to analyze the sign of *DS*', which is the combination effect of the simplified system. Meanwhile, with the sensitivity information of intermediate process to inhibitors, we can compare the *DS *values of different inhibitors that have the same structure properties and select proper drug combinations with synergism property.

### Methods for the case study of TNF-α-induced NFκB pathway

#### Model construction and drug selection (see details in Results and Discussion)

We constructed the TNF-α-induced NFκB pathway in Human umbilical vein endothelial cell (HUVEC). The model was developed based on literatures [23-26]. We tweaked the parameter values in terms of the experimental data derived from HUVEC [27]. The new pathway model yielded a better simulation of NFκB activation in HUVEC. The details of Ordinary Differential Equations (ODEs) model of the NFκB pathway could be found in the Additional file 2.

According to simulation results, we made a short list of NFκB activity inhibitors covering three key nodes in the pathway including Proteasome Inhibitor II Aldehyde, HSP90 inhibitor Geldanamycin and IKK-β inhibitor PS-1145 (Additional file 3, Table S5). To determine the doses of these inhibitors in our experiment, we refer to the relative IC50 values of these inhibitors taken from the published experimental and clinical data [28-30]. In our model, Intercellular cell adhesion molecule-1 (ICAM-1) is very sensitive to TNF-α stimulation. It is directly regulated by activated NFκB and become output index of downstream of this pathway.

In the simulation, the changes on relative reaction velocity constants were taken as the inhibition influence on the targets [31,32]. According to Lemma 1, we simplified the system and considered the synergism assessment factor on this simplified system. Through simulations with changing the reaction velocity constants in a wide range that responded to a wide dose range, the drug combination effects of the simplified system were gained. Additional file 3 provides a detailed description of the simulation algorithm.

#### Cell culture and reagents

To evaluate the computational results of our method, we conducted a cell experiment as follows. HUVECs were isolated from freshly obtained human umbilical cords by established methods [33]. The cells were grown onto gelatin-coated 10 cm^2 ^culture dishes in a standard endothelial cell medium (ECM) (ScienCell Research Laboratories). ECM consists of 500 ml of basal medium, 25 ml of fetal bovine serum (FBS), 5 ml of endothelial cell growth supplement (ECGS) and 5 ml of penicillin/streptomycin solution (P/S). Cells used for this study were from passages 4 to 8 in ECM at 37°C in a 5% CO_2_/humidified air incubator and starved for 6 hours in 0.1% FBS medium before each assay. All experiments were carried out when the cells were 80% confluent. Proteasome Inhibitor II Aldehyde and HSP90 inhibitor Geldanamycin were purchased from Alexis Biochemicals (San Diego, CA). IκB kinase (IKK-β) inhibitor PS-1145 was from Sigma-Aldrich (St. Louis, MO). These inhibitors were dissolved in DMSO and stored at -20°C until use. The recombinant human TNF-α (rh-TNF-α) protein was purchased from Cell Signaling Technology Inc. (CST, Beverly, MA). Antibodies to ICAM-1 and β-actin were obtained from CST.

#### Western blotting

To detect the effect of three combinations on output index of this pathway, ICAM-1 directly regulated by NFκB was investigated by western blotting as follows. HUVECs were treated with 100 nM Aldehyde, 100 nM Geldanamycin, 100 nM PS-1145 and various combinations of Aldehyde, Geldanamycin and PS-1145 at the dose of 100 nM for 2 hours followed by 10 ng/ml TNF-α. After 6 hours of treatment, whole-cell extracts from treated cells and immunoblotting were prepared as previously described [34]. Whole cell lysates were subjected to SDS-PAGE 10% gels. Proteins were transferred to nitrocellulose blotting membranes (Bio-Rad Laboratories, Hercules, CA), and immunoblotted 4°C overnight with anti-ICAM-1 and anti-β-actin Abs (typically 1:1,000 dilution) followed by secondary antibody conjugated with horseradish peroxidase (1:10,000 dilution). The SuperSignal^® ^West Dura (Thermo Fisher Scientific, Rockford, Ill, USA) was used for detection according to the manufacturer's instruction.

## Results and Discussion

Most signaling pathways are constructed from three types of structures - serial, parallel and feedback, so our study will be focusing on these three structures. Based on Lemma 1, the simplifications of serial structures and parallel structures are studied. We also analyze the influence of feedback structure on serial structures. Numerical examples are also given; the simulation results of both original and simplified systems showed potency of the method on analyzing the combination effects. All these structures and figures are from Fitzgerald's work [1].

### Simplification rule for serial structure

**Figure 1:**
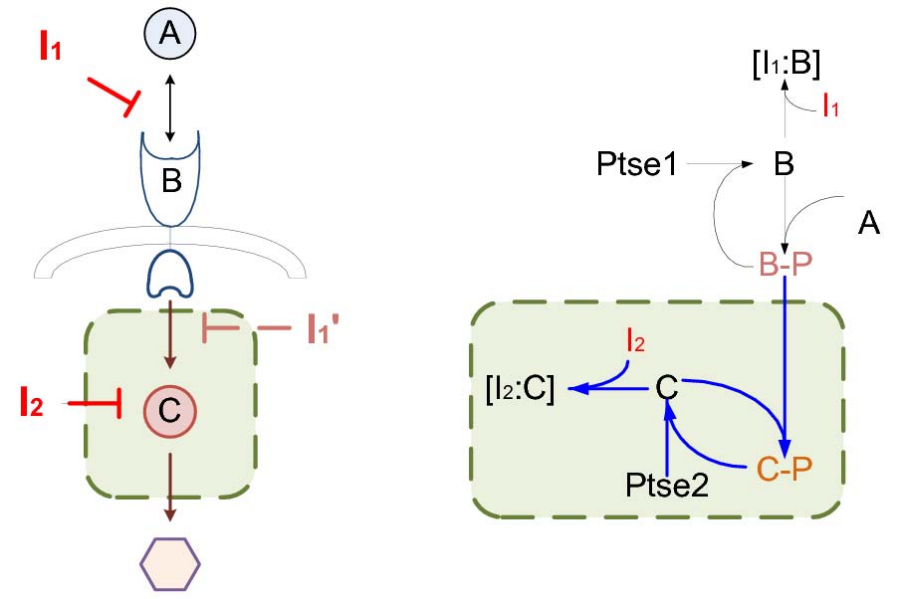
**Serial structure**. The left subfigure illustrate the general serial structure. The right subfigure is an example from [1]. The shaded areas are the simplified systems. Based on the simplification rules the virtual inhibitors on the simplified systems in this example target on the reaction B-P+C→C-P (or it also could be seen as target on component B-P) and component C.

In Figure 1, A activates B, then B activates C. The concentration of activated C is the output of this system. The sequential activation processes construct a typical serial structure. To illustrate the drug combination effects, we designate that I_1 _and I_2 _are inhibitors affecting the two activation processes separately.

#### Corollary 1

The sign of the synergism assessment factor derivative *DS *of original serial structure in Figure 1 is opposite to the sign of the synergism assessment factor derivative *DS' *of the simplified structure (shaded area in Figure 1). That is to say,

### Simplification rule for parallel structure

**Figure 2:**
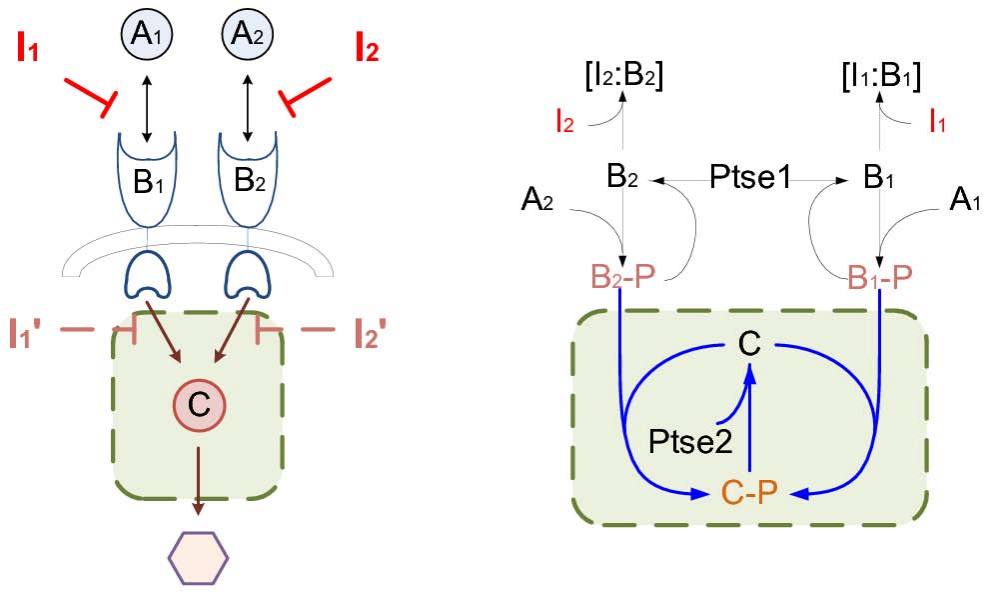
**Parallel structure**. The left subfigure illustrate the general parallel structure. The right subfigure is an example from [1]. The shaded areas are the simplified systems. Based on the simplification rules the virtual inhibitors on the simplified systems in this example target on the reactions B_1_-P+C→C-P (or it also could be seen as target on component B_1_-P) and B_2_-P+C→C-P (or target on component B_2_-P).

In Figure 2, there are parallel activation processes: A_1 _activates B_1_, A_2 _activates B_2_. As in serial structure, I_1 _and I_2 _are inhibitors affecting those two activation processes separately. Both B_1 _and B_2 _can activate C. The output of this system is the concentration of activated C. The relation between B_1 _and B_2 _could be demonstrated as logic OR.

#### Corollary 2

The sign of the synergism assessment factor derivative *DS *of original parallel structure in Figure 2 is the same as the sign of the synergism assessment factor derivative *DS' *of the simplified structure (shaded area in Figure 2). That is to say,

By Corollary 1 and 2, whether the simplified systems could generate synergism under equivalent inhibition can help to determine whether the original systems can generate synergism effect under actual inhibition (see details of proofs in Additional file 1). In Figure 1, the original serial structure under I_1 _and I_2 _inhibition can be simplified as the system (shaded) under equivalent I_1' _and I_2 _inhibition from the view of combination effects; It is the same to simplify the original parallel structure under I_1 _and I_2 _inhibition as a system under equivalent I_1' _and I_2' _inhibition. This could simplify the system structures, meanwhile simplify the drug combination analysis. Besides, it becomes easier to find drug combinations that could generate synergism on the systems based on these conclusions.

### Combination effect preservation rule for systems with feedback

Feedback structures are common regulatory structures in biological systems, especially in signaling pathways. Positive and negative feedback structures could shape the signaling responses in time and space [35], like performancing as oscillators or bistable switches. Feedback structures increase the complexity of system structures, and make it more difficult to analyze the drug combination effects on the systems. From the viewpoint of drug combination therapy, sometimes we just need to know how the feedback structures influence the drug combination effects.

**Figure 3:**
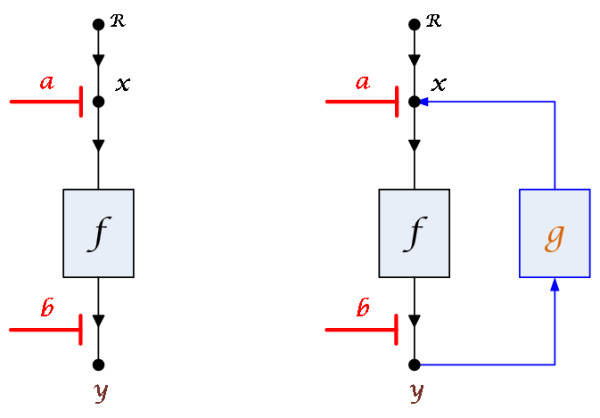
**Illustration of feedback structures**. *R *is the input of *x*. *y *is the output of the system. *f *is the intermediate process from *x *to *y*. *g *is the feedback function. *a *and *b *are affected parameters.

Considering an original system without feedback loop that can generate synergism effect under some drug combinations (left subfigure in Figure 3), if after adding a feedback loop (right subfigure in Figure 3), the output of the new system decreases compared to that of original system, then the feedback loop can be believed that it strengthens the drug combination effects. Upon that, it could only consider combination effect analysis on the original system.

In general feedback structures (as shown in right subfigure in Figure 3) and the system without the feedback loop can be modeled by Ordinary Differential Equations (ODEs) separately as

#### Lemma 2

Adding feedback loop to a system will not decrease the drug combination effects of the original system if . That is,

1) Negative feedback **(***g*(·) <**0)**, and  >**0**

or

2) Positive feedback **(***g*(·) <**0)**, and  <**0 **is satisfied. Details of proof are given in Additional file 4.

### Examples of serial structure and parallel structure

**Figure 4:**
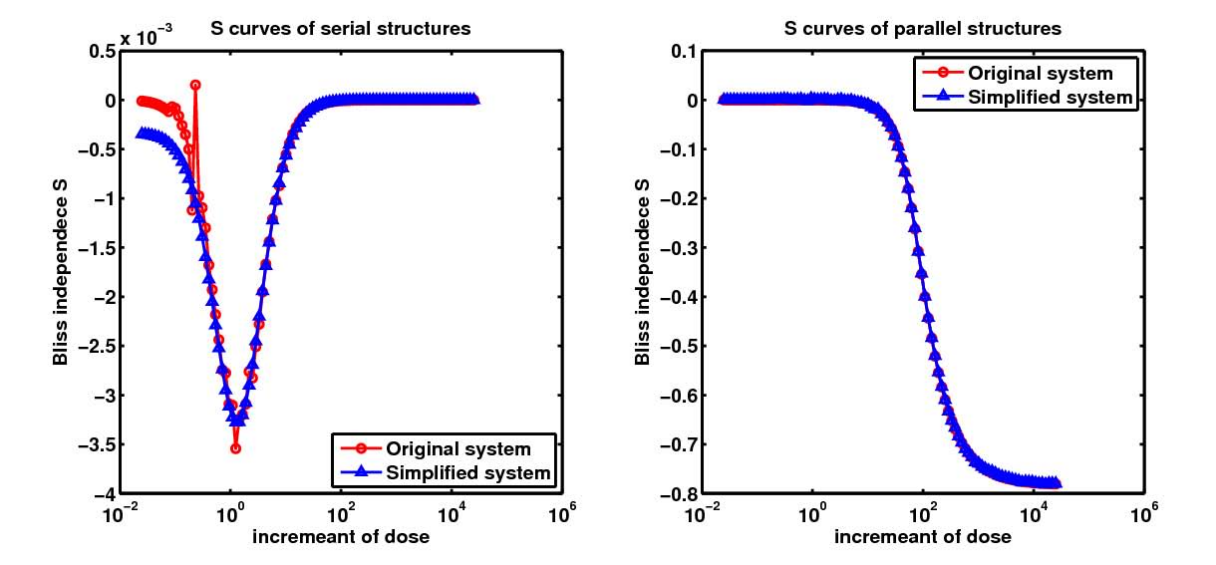
***S *curves of Figure 1 and 2**. In the simulation, we adopted the same doses of both inhibitors like [1]. The left subfigure is the simulation results of the serial structure in Figure 1. The right subfigure is the simulation results of the parallel structure in Figure 2. The *S *curves of original systems are in red. The blue curves are the *S *curves recovered from the results of simplified systems based on Lemma 1 and the two corollaries. In fact the *S *curve of the simplified serial system has opposite sign to that of the original serial system. Obviously the *S *curves recovered agreed with the original *S *curves. The signs of *S *curves are negative showing the synergistic effect of the serial and parallel structures. In left subfigure the absolute values of *S *are much small (10^-3 ^order) to those of the *S *in right subfigure (10^-1 ^order). Combination therapy of the parallel structure could be more effective. These results are conincident with the results in [1].

We adapted numerical examples in [1] as shown in Figure 1 and Figure 2 respectively to verify the Corollary 1 and 2 above. All parameters are referred to [1]. In addition, all the reactions are modeled as Michaelis-Menten equations. Direct evaluation of the synergism asscessment factor *S *by simulation leads to the results shown in Figure 4.

In Figure 4, left figure shows the results of serial structure and right figure shows the results of parallel structure. The blue curves referred to the *S *curves recovered from results of the simplified systems, while the red lines referred to the results of the original systems. It is obvious that the recovered *S *curves agreed with the original *S *curves. Actually, the S curve of the simplified serial system has opposite sign to that of the original serial system.

*S *curves with negative signs indicate that both the original serial and parallel structures generate synergistic effect. In left subfigure the absolute values of *S *are smaller (10^-3 ^order) than those of the *S *in right subfigure (10^-1 ^order). Combination therapy of the parallel structure could be more effective. These results are conincident with the results in [1]. As a comparison from these results, the new method enables us to evaluate the combination effects of original systems by analyzing the effects of the corresponding simplified systems.

### An example of negative feedback structure

Figure 5 from [1] shows the example of negative feedback structure. This negative feedback system is constructed based on the serial system in Figure 1. From the results in [1] the influence of feedback on combination effects is verified to have nonnegative impact under the condition 1) in Lemma 2.

**Figure 5:**
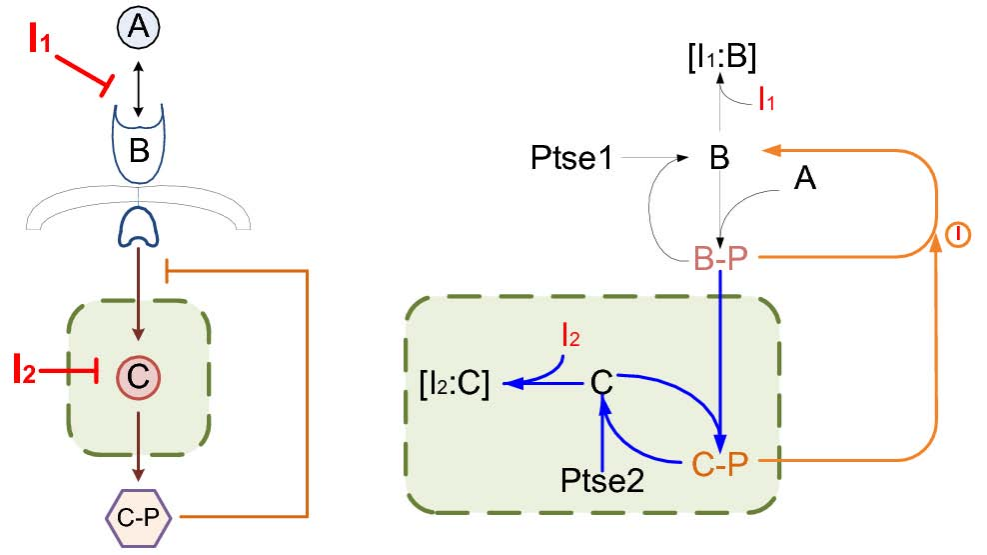
**Negative feedback structure**. This system with negative feedback loop (from [1]) is constructed based on the serial system in Figure 1 by adding a feedback loop (orange color) which is from the output C-P to the activation of B to the serial structure. The output of this system is still the concentration of activation C.

Based on the ODEs of the serial structure, the ODEs of this feedback structure are as follows:

In the model, *x*_1 _and *x*_2 _are concentrations of B-P and C-P (activated C) respectively. *x*_2 _is the output of the system. *x*_1*t *_and *x*_2*t *_are initial concentrations of B and C respectively. *R *is the concentration of A. *K*_*m*1 _and *K*_*m*2 _are Michaelis-Menten constants of the B→B-P and C→C-P activation and are affected by I_1 _and I_2_, respectively. *K*_*m*5 _are Michaelis-Menten constants of B-P→B activation mediated by C-P. From the simulation results in [1], it is obvious that the output of feedback structure example is smaller than the output of serial structure. This comparison verified Lemma 2 that feedback loop can strengthen the drug combination effects.

It should be pionted out, although our method leads to simplified systems, that does not mean the analysis of the simplified systems is easy. Usually simulations or experiments are still needed for analyzing these systems due to their complex dynamics [14,36-38]. Our method also relies on the faithful modeling of the systems which may not be trivial since identifying system structure in general is still a challenging work [39-41].

### Results and discussion for the case study of TNF-α-induced NFκB pathway

**Figure 6:**
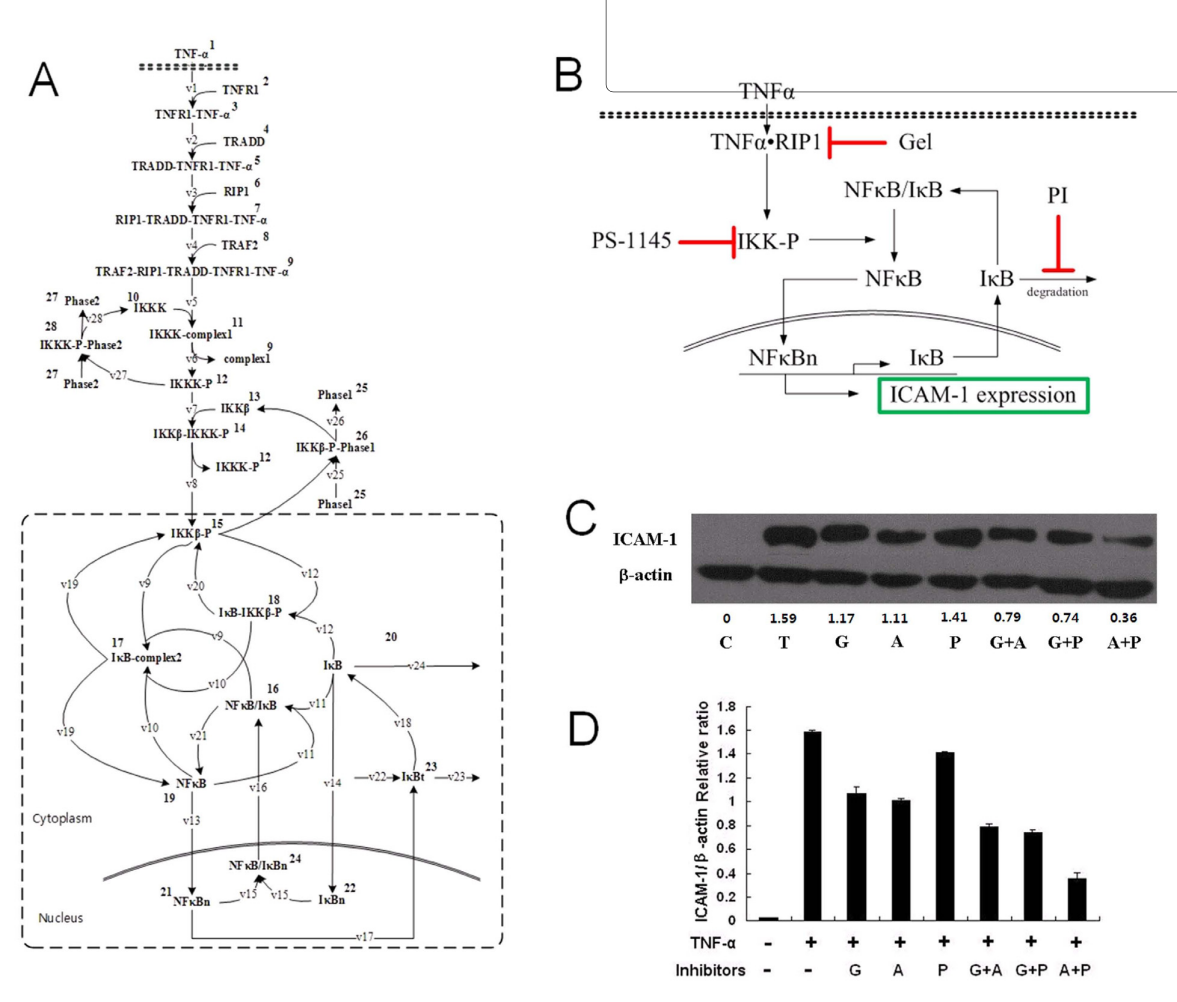
**Case study of TNF-α-induced NFκB pathway**. A. The kinetic model of TNF-α-induced NFκB pathway. The dashed box area indicates the simplified system. B. Biological schematic of TNF-α-induced NFκB pathway. Not all arrows represent direct physical interactions. Drugs used in this study and their key targets are highlighted. C. HUVECs were pretreated with three combinations at the same concentration 100 nM for 2 hours before being stimulated with 10 ng/ml TNF-α for 6 hours. Cells were lysed and output signaling protein was probed by western blotting. The level of β-actin was used as an internal control for normalization. The gray values are recorded at the bottom. D. Ratio of ICAM-1 to β-actin was determined with densitometry. C, control, T, 10 ng/ml TNF-α, G, Geldanamycin, A, Aldehyde, P, PS-1145.

TNF-α is both a pro-inflammation cytokine and a pro-angiogenic factor [42]. It is responsible for inflammatory angiogenesis and tumorigenesis. The induction of NFκB signaling pathway by TNF-α can regulate the transcriptional expression of several genes in vascular endothelial cells that lead to angiogenesis [42]. Several standard drug targets such as HSP90 and IKK-β are among the key molecules involved in the pathway responsible for generating angiogenic factors. However, essentially all single-target inhibitors have low therapeutic effects in inflammatory and angiogenic diseases. To find more efficient drug combination solutions, we constructed the model of an inflammatory angiogenesis-related pathway, the TNF-α-induced NFκB pathway (Figure 6A) and employed our method in this paper to search for synergistic drug combination solutions within this pathway model.

As a result of our method, IκB degradation proteasome, HSP90 and IKK-β as important targets in the TNF-α-induced NFκB pathway are selected. In the simulation, changes on the relative reaction velocity constants were taken as the inhibition influence on targets. It should be pointed out that Geldanamycin as a HSP90 inhibitor dose not target RIP1 directly but could indirectly regulate the binding rate of RIP1, so we changed the reaction velocity constants of RIP1 binding with TNFR1 complex. The change ratio of the reaction velocity constants ranged from 0.9 to 0.0001 fold to cover a wide dose range.

As shown in Figure 6B, the TNF-α-induced NFκB pathway has a serial structure with feedback. The three targets locate on the serial path (RIP1 and IKK-β) and feedback path (IκB degradation), especially IKK-β is on the joint of serial path and feedback path. Since TRAF2-RIP1-TRADD-TNFR1-TNF-α activates IKK-β, the inhibition effect on RIP1 could be seen as finally acted on IKK-β. According to Lemma 1, the system could be simplified as the dashed box area in Figure 6A. The corresponding reactions inhibited by the three inhibitors in original and simplified systems were shown in Table 1. We then employed our method to calculate the synergism assessment factor on the simplified system. The sign of synergism assessment factor decides whether there is synergistic effect or not. The value of synergism assessment factor reflects the efficacy extent of drug combination from Aldehyde, Geldanamycin and PS-1145 (shown in Figure 6B). We screened out the synergistic drug combinations according to the sign of synergism assessment factor in simplified system. Then we did the simulation on the original system for the synergistic drug combinations. The simulation results are shown in Table 2. It shows that all the three drug combinations could generate synergistic effect. And the signs of synergism assessment factors in simplified system were consistent with those of the synergism assessment factors in original system.

**Table 1:** Comparison of reactions inhibited by drugs in original and simplified systems.

Inhibitors	Corresponding reactions in original system	Corresponding reactions in simplified system
Geldanamycin	RIP1 binding with TNFR1 complex	IKKK-P activates IKK-β
PS-1145	IKK-β-P phosphorylates IκB	IKK-β-P phosphorylates IκB
Aldehyde	IκB degradation	IκB degradation

In the simulation, we simplified the system according to Lemma 1. Here we list the corresponding reactions inhibited by the three inhibitors in original and simplified systems.

**Table 2:** Simulation and experimental results for the case study of TNF-α-induced NFκB pathway.

Inhibitor A	Inhibitor B	Synergism Assessment Factor (Simulation)		Predicted interaction	Synergism Assessment Factor (Experiment)
				
		simplified system	original system		
Geldanamycin	Aldehyde	-0.0632	-0.2678	Synergism	-0.0167
Geldanamycin	PS-1145	-0.1248	-0.2555	Synergism	-0.1878
PS-1145	Aldehyde	-0.1754	-0.1754	Synergism	-0.3931

Here we consider the synergism assessment factors of the three combinations in the simplified and original system. The sign of synergism assessment factor indicates whether the drug combination generates synergistic effect or not. If the sign is negative, it is synergistic effect. Otherwise, it is antagonistic effect. In the experiment, ICAM-1 expression is considered as the measurement readout of the efficacy of drugs or drug combinations. We also evaluate the effects of drug combination by calculating synergism assessment factor (see Eq. 3). The negative values represent synergism since we rewrite calculation formula of Bliss independence model according to survival ratio rather than inhibition ratio.

ICAM-1, an intercellular adhesion molecule expressed in endothelial cells, is a common cellular readout of TNF-α induced signaling pathway [43]. To verify the simulation results, we observed the effect of Aldehyde, Geldanamycin, PS-1145 and related combinations on ICAM-1 expression level by western blotting analysis. In the experiment, we used Bliss independent model to assess whether three combinations induced synergistic inhibition effect on ICAM-1 expression. As shown in Figure 6C and Figure 6D, all the three drug combinations generate synergistic effect. Taken together, this result suggests that the synergistic combinations predicted by our method are qualitatively consistent with the experimental observations. Currently, the method can only make qualitative prediction on drug combinational effects. This still could provide some clues for drug combination design based on mechanisms. Besides, as the model we provided here is specific for TNF-α-induced NFκB pathway in HUVEC, the general applicability of our method still needs further investigation.

## Conclusions

In this paper we presented a new method based on an extended Bliss independence criterion to analyze the relationship between structures and effects for combination drug targets design from a mathematical aspect. We analyzed two classic structures, serial structure and parallel structure, and showed in steady state the sign of the synergism assessment factor derivative of the original system can be predicted by the sign of its simplified system. In addition, we analyzed the influence of feedback structure on survival ratio of the system, and showed that the feedback structure could not destroy the drug combination effect of the system without feedback under some conditions. We demonstrated by numerical examples that these results are useful for reducing the amount of computatioal load if system reaction network topology knowledge is available. In the case study, the effects of inhibitor combinations predicted by our method were experimentally validated by measuring the output (ICAM-1 expression) of TNF-α-induced NFκB pathway. Hopefully, this work can provide some insights to tackle the challenging problem of assessment of combination drug therapy effct in a large scale signaling pathways. As we point out, Bliss model is relatively simple, so in this paper we focused on this simple model. With deep understanding of dose-effect curves, we hope in the future our method could be expanded to more general criteria such as the law of mass action [10-12,21].

## Authors' contributions

HY: study conception, research design, mathematical analysis, manuscript writing; BZ: model constructing, experimental study, manuscript writing; SL: directed the design of the model constructing, experimental study, study conception, helped with the bioinformatics analyses, participated the revision of the manuscript; QZ: formulated and directed the design of the study. All authors read and approved the final manuscript.

## Note

In the following figures (Figure 1, 2, 3, 4, 5), A, B, C, A_1_, A_2_, B_1_, B_2 _are components of the systems, I_1 _and I_2 _are inhibitors. The shaded areas represent simplified systems for the original systems. I_1' _and I_2' _are virtual inhibitors on the simplified systems equivalent to I_1 _and I_2 _.

## Supplementary Material

Additional file 1 **Proof of system simplification rules**. Proof of Lemma 1 (fundamental property of synergism assessment factor derivative), Corollary 1 (simplification rule for serial structure) and 2 (simplification rule for parallel structure). Click here for file

Additional file 2 **Summary of the model of TNF-α-induced NFκB pathway**. Tables S1 - S4 list details of the mathematical model of TNF-α-induced NFκB pathway including reactions and rate equations, initial components concentrations, kinetic parameters and ordinary differential equations. Click here for file

Additional file 3 **Summary of algorithm for TNF-α-induced NFκB pathway**. Implementation of the algorithm for TNF-α-induced NFκB pathway. Click here for file

Additional file 4 **Proof of influence of feedback**. Proof of Lemma 2 for influence of feedback structure to original serial system based on comparison principle that is used to compute bounds on solutions of differential equations. Click here for file
