# Supplementary material for: A formal model for analyzing drug combination effects and its application in TNF-α-induced NFκB pathway
Source: BMC Syst Biol. 2010 Apr 25;4:50. doi: 10.1186/1752-0509-4-50 (PMC2873319; doi:10.1186/1752-0509-4-50)
Supplement: Additional file 1 — Proof of system simplification rules. Proof of Lemma 1 (fundamental property of synergism assessment factor derivative), Corollary 1 (simplification rule for serial structure) and 2 (simplification rule for parallel structure). [file 1752-0509-4-50-S1.DOC]

## Additional file 1 – Proof of system simplification rules

**Lemma 1:** $a$ and $b$ are system parameters that will be affected by inhibitors. $a$ is of processes that will produce product $x$($x=\Phi(a)$), and $b$ is of processes that will produce product $y$($y=\Psi(b)$). Then synergism assessment factor derivative $DS$ of the original system satisfies

$$DS=DS'\frac{\partial x}{\partial a}\frac{\partial y}{\partial b}$$

Where $DS'=\frac{\partial^{2}r(x,y)}{\partial x\partial y}-\frac{\partial r(x,y_{0})}{x}\frac{\partial r(x_{0},y)}{\partial y}$,$x=\Phi(a)$,$y=\Psi(b)$,$x_{0}=\Phi(a_{0})$, $y_{0}=\Psi(b_{0})$.

**Proof** According to the definition of $DS$,

$$DS=\frac{\partial^{2}r(\Phi(a),\Psi(b))}{\partial a\partial b}-\frac{\partial r(\Phi(a),\Psi(b_{0}))}{\partial a}\frac{\partial r(\Phi(a_{0}),\Psi(b))}{\partial b}$$

Since $x$ and $y$ are functions of $a$ and $b$ separately, $x=\Phi(a)$ and $y=\Psi(b)$,

$$DS=\frac{\partial^{2}r(\Phi(a),\Psi(b))}{\partial\Phi(a)\partial\Psi(b)}\frac{\partial\Phi(a)}{\partial a}\frac{\partial\Psi(b)}{\partial b}$$

$$-(\frac{\partial r(\Phi(a),\Psi(b_{0}))}{\partial\Phi(a)}\frac{\partial\Phi(a)}{\partial a})(\frac{\partial r(\Phi(a_{0}),\Psi(b))}{\partial\Psi(b)}\frac{\partial\Psi(b)}{\partial b})$$

$$=[\frac{\partial^{2}r(\Phi(a),\Psi(b))}{\partial\Phi(a)\partial\Psi(b)}-\frac{\partial r(\Phi(a),\Psi(b_{0}))}{\partial\Phi(a)}\frac{\partial r(\Phi(a_{0}),\Psi(b))}{\partial\Psi(b)}]$$

$$\cdot\frac{\partial\Phi(a)}{\partial a}\frac{\partial\Psi(b)}{\partial b}$$

$$=[\frac{\partial^{2}r(x,y)}{\partial x\partial y}-\frac{\partial r(x,y_{0})}{x}\frac{\partial r(x_{0},y)}{\partial y}]\frac{\partial x}{\partial a}\frac{\partial y}{\partial b}$$

$$=DS'\frac{\partial x}{\partial a}\frac{\partial y}{\partial b}$$

where $x=\Phi(a)$,$y=\Psi(b)$,$x_{0}=\Phi(a_{0})$, $y_{0}=\Psi(b_{0})$. (#)

**Corollary 1** The sign of the synergism assessment factor derivative *DS* of original serial structure in Figure 1 is opposite to the sign of the synergism assessment factor derivative *DS'* of the simplified structure (shaded area in Figure 1). That is to say,

$$sign(DS)=-sign(DS')$$

**Proof** Following Michaelis-Menten equations, the ODEs model of serial structure is

$$a =K_{m1}$$

$$b =K_{m2}$$

In the model, $x_{1}$ and $x_{2}$ are concentrations of B-P and C-P respectively, $x_{2}$ is the output of the system. $x_{1t}$ and $x_{2t}$ are initial concentrations of B and C respectively. *R* is concentration of A. $K_{m1}$ and $K_{m2}$ are Michaelis constants of the BB-P and CC-P activation and are affected by I_1_ and I_2_ respectively.

The survival ratio $r(a,b)=\frac{x_{2}(a,b)}{x_{2}(a_{0},b_{0})}$, where $a_{0}$, $b_{0}$ is the values of $K_{m1}$ and $K_{m2}$ before inhibition.

According to Lemma 1

$$DS=DS'\frac{\partial\Phi(a)}{\partial a}\frac{\partial\Psi(b)}{\partial b}$$

$$=DS'\frac{\partial\Phi(a)}{\partial a}$$

where $\Phi(a)=x_{1}(a)$, $\Psi(b)=b$, $\Phi(a_{0})=x_{1}(a_{0})$, $\Psi(b_{0})=b_{0}$. Then the sign of *DS* is determined by the sign of *DS'* and the sign of $\frac{\partial\Phi(a)}{\partial a}$**.**

The steady state equation of $x_{1}$ ($a$ stands for $K_{m1}$) is . According to the Goldbeter and Koshland function,

$G(u,v,J,K)$ $=\frac{2uK}{v-u+vJ+uK+\sqrt{(v-u+vJ+uK)^{2}-4(v-u)uK}}$ $=\frac{C}{A+\sqrt{A^{2}-B}}$ (A.1)

we can get the steady state of $x_{1}$:

$x_{1}=x_{1t}G(k_{1}R,V_{1},\frac{a}{x_{1t}},\frac{K_{m3}}{x_{1t}})$ (A.2)

The sign of $\frac{\partial x_{1}}{\partial a}$ (that is $\frac{\partial\Phi(a)}{\partial a}$) is easy to know:

$$\frac{\partial x_{1}}{\partial a}=-\frac{x_{1t}C}{(A+\sqrt{A^{2}-B})^{2}}(1+\frac{A}{\sqrt{A^{2}-B}})\frac{\partial A}{\partial a}$$

$$=-\frac{x_{1}}{\sqrt{A^{2}-B}}\frac{V_{1}}{x_{1t}}<0$$

Then the sign of $DS$is

$sign(DS)=-sign(DS')$ (#)

**Corollary 2** The sign of the synergism assessment factor derivative *DS* of original parallel structure in Figure 2 is the same as the sign of the synergism assessment factor derivative *DS'* of the simplified structure (shaded area in Figure 2). That is to say,

$$sign(DS)=sign(DS')$$

**Proof** Proof is almost the same as proof of Corollary 1.

Following Michaelis-Menten equations, the ODEs model of serial structure is

$$a =K_{m1}$$

$$b =K_{m2}$$

In the model, $x_{1}$ and $x_{2}$ are concentrations of B_1_-P and B_2_-P respectively. $x_{3}$ is concentration of C-P, the output of the system. $x_{1t}$ , $x_{2t}$ and $x_{3t}$ are initial concentrations of B_1_, B_2_ and C respectively. *R*_1_ and *R*_2_ are concentrations of A_1_ and A_2_. $K_{m1}$ and $K_{m2}$ are Michaelis constants of the B_1_B_1_-P and B_2_B_2_-P activation and are affected by $I_{1}$ and $I_{2}$ respectively. $K_{m5}$ and $K_{m6}$ are Michaelis constants of CC-P activation.

The survival ratio $r(a,b)=\frac{x_{3}(a,b)}{x_{3}(a_{0},b_{0})}$, where $a_{0}$, $b_{0}$ is the values of $K_{m1}$ and $K_{m2}$ before inhibition.

According to Lemma 1

$$DS=DS'\frac{\partial\Phi(a)}{\partial a}\frac{\partial\Psi(b)}{\partial b}$$

where $\Phi(a)=x_{1}(a)$, $\Psi(b)=x_{2}(b)$, $\Phi(a_{0})=x_{1}(a_{0})$, $\Psi(b_{0})=x_{2}(b_{0})$.

The signs of $\frac{\partial\Phi(a)}{\partial a}$ and $\frac{\partial\Psi(b)}{\partial b}$ can be calculate as follows:

$$\frac{\partial x_{1}}{\partial a}=-\frac{x_{1t}C_{1}}{(A_{1}+\sqrt{A_{1}^{2}-B_{1}})^{2}}(1+\frac{A_{1}}{\sqrt{A_{1}^{2}-B_{1}}})\frac{\partial A_{1}}{\partial a}$$

$$=-\frac{x_{1}}{\sqrt{A_{1}^{2}-B_{1}}}\frac{V_{1}}{x_{1t}}<0$$

$$\frac{\partial x_{2}}{\partial b}=-\frac{x_{2t}C_{2}}{(A_{2}+\sqrt{A_{2}^{2}-B_{2}})^{2}}(1+\frac{A_{2}}{\sqrt{A_{2}^{2}-B_{2}}})\frac{\partial A_{2}}{\partial b}$$

$$=-\frac{x_{2}}{\sqrt{A_{2}^{2}-B_{2}}}\frac{V_{2}}{x_{2t}}<0$$

Then $\frac{\partial\Phi(a)}{\partial a}\frac{\partial\Psi(b)}{\partial b}=\frac{\partial x_{1}}{\partial a}\frac{\partial x_{2}}{\partial b}>0$.

The sign of $DS$is

$sign(DS)=sign(DS')$ (#)
