# Supplementary material for: A formal model for analyzing drug combination effects and its application in TNF-α-induced NFκB pathway
Source: BMC Syst Biol. 2010 Apr 25;4:50. doi: 10.1186/1752-0509-4-50 (PMC2873319; doi:10.1186/1752-0509-4-50)
Supplement: Additional file 2 — Summary of the model of TNF-α-induced NFκB pathway. Tables S1 - S4 list details of the mathematical model of TNF-α-induced NFκB pathway including reactions and rate equations, initial components concentrations, kinetic parameters and ordinary differential equations. [file 1752-0509-4-50-S2.DOC]

## Additional file 2 – Proof of influence of feedback

Lemma 2 could be deduced directly from comparison principle which is used to compute bounds on solutions of differential equations and is important in nonlinear systems.

**Comparison Lemma [1]** Consider the scalar differential equation

Where $f(t,u)$is continuous in *t* and locally Lipschitz in *u*, for all $t\geq0$ and all $u\in J\subset R$. Let $[t_{0},T)$ (*T* could be infinity) be the maximal interval of existence of the solution $u(t)$, and suppose $u(t)\in J$ for all $t\in[t_{0},T)$. Let $v(t)$ be a continuous function whose upper right-hand derivative $D^{+}v(t)$ satisfies the differential inequality

$$D^{+}v(t)\leq f(t,v(t)),v(t_{0})\leq u_{0}$$

With $v(t)\in J$ for all $t\in[t_{0},T)$. Then, $v(t)\leq u(t)$ for all $t\in[t_{0},T)$.

**Lemma 2:** Adding feedback loop to a system will not decrease the drug combination effects of the original system if $g\cdot\frac{\partial f}{\partial x}<0$. That is,

1. Negative feedback ($g(\cdot)<0$), and $\frac{\partial f}{\partial x}>0$
2. Positive feedback ($g(\cdot)>0$), and $\frac{\partial f}{\partial x}<0$

**Proof** Here are the ODEs of feedback structures and the original serial structures:

| Feedback structures | System without feedback |
| --- | --- |
|  |  |

1. Negative feedback ($g(\cdot)<0$), and $\frac{\partial f}{\partial x}>0$

If $g(\cdot)<0$, then . Since $\frac{\partial f}{\partial x}>0$, . According to Comparison Lemma, $y^{*}(t)<y(t)$.

1. Positive feedback ($g(\cdot)>0$), and $\frac{\partial f}{\partial x}<0$

If $g(\cdot)>0$, then . Since $\frac{\partial f}{\partial x}<0$, . According to Comparison Lemma, $y^{*}(t)<y(t)$.

These mean that the output of system with feedback will decrease compared to that of original system without feedback. Thus, if original system can generate synergism effects under some drug combination scheme, the feedback structure will strengthen the combination effects.

### References

1. Khalil HK: *Nonlinear systems*. 3rd edition. Upper Saddle River, NJ: Prentice Hall; 2002.
