# Supplementary material for: A formal model for analyzing drug combination effects and its application in TNF-α-induced NFκB pathway
Source: BMC Syst Biol. 2010 Apr 25;4:50. doi: 10.1186/1752-0509-4-50 (PMC2873319; doi:10.1186/1752-0509-4-50)
Supplement: Additional file 3 — Summary of algorithm for TNF-α-induced NFκB pathway. Implementation of the algorithm for TNF-α-induced NFκB pathway. [file 1752-0509-4-50-S3.DOC]

## Additional file 3 – Summary of the model of TNF-α-induced NFκB pathway

### Table S1 Reactions and rate equations

| **v** | **Reactions** | **Description** | **Rate equations** | **References** |
| --- | --- | --- | --- | --- |
| 1 | TNF-α + TNFR1 <-> TNFR1-TNF-α | TNF-α-TNFR1 association / disassociation | v(1) = k(1)*c(1)*c(2) - kd(1)*c(3); | (3) |
| 2 | TNFR1-TNF-α + TRADD <-> TRADD-TNFR1-TNF-α | TRADD-TNFR1-TNF-α association / disassociation | v(2) = k(2)*c(3)*c(4) - kd(2)*c(5); | (3) |
| 3 | TRADD-TNFR1-TNF-α + RIP1 <-> RIP1-TRADD-TNFR1-TNF-α | RIP1-TRADD-TNFR1-TNF-α association / disassociation | v(3) = k(3)*c(5)*c(6) - kd(3)*c(7); | (2)(1) |
| 4 | RIP1-TRADD-TNFR1-TNF-α + TRAF2 <-> TRAF2-RIP1-TRADD-TNFR1-TNF-α | TRAF2-RIP1-TRADD-TNFR1-TNF-α association / disassociation | v(4) = k(4)*c(7)*c(8) - kd(4)*c(9); | (3) |
| 5 | TRAF2-RIP1-TRADD-TNFR1-TNF-α + IKKK <-> IKKK-complex1 | IKKK-complex1 association / disassociation | v(5) = k(5)*c(9)*c(10) - kd(5)*c(11); | (3) |
| 6 | IKKK-complex1 -> TRAF2-RIP1-TRADD-TNFR1-TNF-α + IKKK-P | IKKK-complex1 catalysis | v(6) = kd(6)*c(9)*c(12) - k(6)*c(11); | (3) |
| 7 | IKKK-P + IKK-β <-> IKKβ-IKKK-P | IKK-β-IKKK-P association / disassociation | v(7) = k(7)*c(12)*c(13) - kd(7)*c(14); | (3) |
| 8 | IKK-β-IKKK-P -> IKKK-P + IKK-β-P | IKK-β-IKKK-P catalysis | v(8) = kd(8)*c(12)*c(15) - k(8)*c(14); | (3) |
| 9 | IKK-β-P + NFκB/IκB <-> IκB-complex2 | IKK-β-P + NFκB/IκB association / disassociation | v(9) = k(9)*c(15)*c(16) - kd(9)*c(17); | (3) |
| 10 | NFκB + IκB-IKK-β-P <-> IκB-complex2 | NFκB + IκB-IKK-β-P association / disassociation | v(10) = k(10)*c(18)*c(19) - kd(10)*c(17); | (3) |
| 11 | NFκB + IκB <-> NFκB/IκB | NFκB + IκB association / disassociation | v(11) = k(11)*c(19)*c(20) - kd(11)*c(16); | (3) |
| 12 | IκB + IKK-β-P <-> IκB-IKK-β-P | IκB + IKK-β-P association / disassociation | v(12) = k(12)*c(15)*c(20) - kd(12)*c(18); | (3) |
| 13 | NFκB <-> NFκBn | NFκB nuclear import / export | v(13) = k(13)*c(19)*1 - kd(13)*c(21); | (3) |
| 14 | IκB <-> IκBn | IκB nuclear import / export | v(14) = k(14)*c(20)*1 - kd(14)*c(22); | (3) |
| 15 | NFκBn + IκBn <-> NFκB/IκBn | NFκBn + IκBn association / disassociation | v(15) = k(15)*c(21)*c(22) - kd(15)*c(24); | (3) |
| 16 | NFκB/IκBn -> NFκB/IκB | NFκB/IκBn nuclear export | v(16) = k(16)*c(24)*1 - kd(16)*c(16); | (3) |
| 17 | NFκB -> IkBt | IκB inducible mRNA synthesis | v(17) = k(17)*c(21)*c(21) - kd(17)*c(23); | (3) |
| 18 | IκBt -> IκB | constitutive IκB translation | v(18) = k(18)*c(23)*1 - kd(18)*c(20); | (3) |
| 19 | IκB-complex2 -> NFκB + IKK-β-P | IκB-complex2 catalysis | v(19) = kd(19)*c(15)*c(19) - k(19)*c(17); | (3) |
| 20 | IκB-IKK-β-P -> IKK-β-P | IκB-IKK-β-P catalysis | v(20) = k(20)*c(18)*1 - kd(20)*c(15); | (3) |
| 21 | NFκB/IκB -> NFκB | constitutive IκB degradation (bound) | v(21) = k(21)*c(16)*1 - kd(21)*c(19); | (3) |
| 22 | -> IκBt | IκB constitutive mRNA synthesis | v(22) = k(22)*1*1 - kd(22)*c(23); | (3) |
| 23 | IκBt -> | IκB mRNA degradation | v(23) = k(23)*c(23)*1 - kd(23)*1; | (3) |
| 24 | IκB -> | constitutive IκB degradation (free) | v(24) = k(24)*c(20)*1 - kd(24)*1; | (3) |
| 25 | Phase1 + IKK-β-P <-> IKK-β-P-Phase1 | IKK-β-P-Phase1 association / disassociation | v(25) = k(25)*c(15)*c(25) - kd(25)*c(26); | (3) |
| 26 | IKK-β-P-Phase1 -> Phase1 + IKK-β | IKK-β-P-Phase1 catalysis | v(26) = kd(26)*c(13)*c(25) - k(26)*c(26); | (3) |
| 27 | Phase2 + IKKK-P <-> IKKK-P-Phase2 | IKKK-P-Phase2 association / disassociation | v(27) = k(27)*c(12)*c(27) - kd(27)*c(28); | (3) |
| 28 | IKKK-P-Phase2 -> Phase2 + IKKK | IKKK-P-Phase2 catalysis | v(28) = kd(28)*c(10)*c(27) - k(28)*c(28); | (3) |

### Table S2 Initial concentrations

| **c** | **Components** | **Initial concentrations (nM)** | **References** |
| --- | --- | --- | --- |
| 1 | TNF-α | 0.588 | (10ng/ml) |
| 2 | TNFR1 | 166.67 | (3) |
| 3 | TNFR1-TNF-α | 0 | (3) |
| 4 | TRADD | 100 | (3) |
| 5 | TRADD-TNFR1-TNF-α | 0 | (3) |
| 6 | RIP1 | 200 | Assume |
| 7 | RIP1-TRADD-TNFR1-TNF-α | 0 | (3) |
| 8 | TRAF2 | 333.89 | (3) |
| 9 | TRAF2-RIP1-TRADD-TNFR1-TNF-α | 0 | (3) |
| 10 | IKKK | 45.22 | (3) |
| 11 | IKKK-complex1 | 0 | (3) |
| 12 | IKKK-P | 0 | (3) |
| 13 | IKK-β | 22.78 | (3) |
| 14 | IKK-β-IKKK-P | 0 | (3) |
| 15 | IKK-β-P | 0 | (3) |
| 16 | NFκB/IκB | 100 | (3) |
| 17 | IκB-complex2 | 0 | (3) |
| 18 | IκB-IKK-β-P | 0 | (3) |
| 19 | NFκB | 0 | (3) |
| 20 | IκB | 0 | (3) |
| 21 | NFκBn | 0 | (3) |
| 22 | IκBn | 0 | (3) |
| 23 | IκBt | 0 | (3) |
| 24 | NFκB/IκBn | 0 | (3) |
| 25 | Phase1 | 11.305 | (3) |
| 26 | IKK-β-P-Phase1 | 0 | (3) |
| 27 | Phase2 | 83.4725 | (3) |
| 28 | IKKK-P-Phase2 | 0 | (3) |

### Table S3 Kinetic parameters

|  | **k** | **Value** | **Unit** | **kd** | **Value** | **Unit** | **References** |
| --- | --- | --- | --- | --- | --- | --- | --- |
| 1 | TNF-α-TNFR1 association | 0.000185 | nM ^-1^·s ^-1^ | TNF-α-TNFR1 disassociation | 0.002 | s ^-1^ | (2), (3) |
| 2 | TRADD-TNFR1-TNF-α association | 0.000185 | nM ^-1^·s ^-1^ | TRADD-TNFR1-TNF-α disassociation | 0.00125 | s ^-1^ | (3) |
| 3 | RIP1-TRADD-TNFR1-TNF-α association | 0.000185 | nM ^-1^·s ^-1^ | RIP1-TRADD-TNFR1-TNF-α disassociation | 0.00125 | s ^-1^ | (1)(2) |
| 4 | TRAF2-RIP1-TRADD-TNFR1-TNF-α association | 0.000185 | nM ^-1^·s ^-1^ | TRAF2-RIP1-TRADD-TNFR1-TNF-α disassociation | 0.00125 | s ^-1^ | (3) |
| 5 | IKKK-complex1 association | 0.01 | nM ^-1^·s ^-1^ | IKKK-complex1 disassociation | 0.5 | s ^-1^ | (3) |
| 6 | IKKK-complex1 catalysis | 0.1 | s ^-1^ |  | 0 |  | (3) |
| 7 | IKK-β-IKKK-P association | 0.01 | nM ^-1^·s ^-1^ | IKK-β-IKKK-P disassociation | 0.5 | s ^-1^ | (3) |
| 8 | IKK-β-IKKK-P catalysis | 0.04 | s ^-1^ |  | 0 |  | refitted |
| 9 | IKK-β-P + NFκB/IκB association | 3.00E-04 | nM ^-1^·s ^-1^ | IKK-β-P + NFκB/IκB disassociation | 0.00125 | s ^-1^ | refitted |
| 10 | NFκB + IκB-IKK-β-P association | 0.0005 | nM ^-1^·s ^-1^ | NFκB + IκB-IKK-β-P disassociation | 0.0005 | s ^-1^ | (3) |
| 11 | NFκB + IκB association | 0.0005 | nM ^-1^·s ^-1^ | NFκB + IκB disassociation | 0.0005 | s ^-1^ | (3) |
| 12 | IκB + IKK-β-P association | 2.25E-05 | nM ^-1^·s ^-1^ | IκB + IKK-β-P disassociation | 0.00125 | s ^-1^ | (3) |
| 13 | NFκB nuclear import | 0.09 | s ^-1^ | NFκB nuclear export | 8E-05 | s ^-1^ | (3) |
| 14 | IκB nuclear import | 0.0003 | s ^-1^ | IκB nuclear export | 0.0002 | s ^-1^ | (3) |
| 15 | NFκBn + IκBn association | 0.0005 | nM ^-1^·s ^-1^ | NFκBn + IκBn disassociation | 0.0005 | s ^-1^ | (3) |
| 16 | NFκB/IκBn nuclear export | 0.0138 | s ^-1^ |  | 0 |  | (3) |
| 17 | IκB inducible mRNA synthesis | 1.67E-05 | nM ^-1^·s ^-1^ |  | 0 |  | (3) |
| 18 | constitutive IκB translation | 6.12E-05 | s ^-1^ |  | 0 |  | refitted |
| 19 | IκB-complex2 catalysis | 0.204 | s ^-1^ |  | 0 |  | refitted |
| 20 | IκB-IKK-β-P catalysis | 0.0407 | s ^-1^ |  | 0 |  | refitted |
| 21 | constitutive IκB degradation (bound) | 2.25E-05 | s ^-1^ |  | 0 |  | (3) |
| 22 | IκB constitutive mRNA synthesis | 1.54E-09 | nM ^-1^·s ^-1^ |  | 0 |  | (3) |
| 23 | IκB mRNA degradation | 0.00028 | s ^-1^ |  | 0 |  | (3) |
| 24 | constitutive IκB degradation (free) | 3.77E-04 | s ^-1^ |  | 0 |  | refitted |
| 25 | IKK-β-P-Phase1 association | 0.01 | nM ^-1^·s ^-1^ | IKK-β-P-Phase1 disassociation | 0.5 | s ^-1^ | (3) |
| 26 | IKK-β-P-Phase1 catalysis | 0.1 | s ^-1^ |  | 0 |  | (3) |
| 27 | IKKK-P-Phase2 association | 0.01 | nM ^-1^·s ^-1^ | IKKK-P-Phase2 disassociation | 0.5 | s ^-1^ | (3) |
| 28 | IKKK-P-Phase2 catalysis | 0.1 | s ^-1^ |  | 0 |  | (3) |

### Table S4 ODEs in the computational model

Here, "dc" means the derivative of concentrations (c), which is the concentration change rate.

| **dc** | **ODEs** |
| --- | --- |
| 1 | dc(1) = 0; |
| 2 | dc(2) = -v(1); |
| 3 | dc(3) = v(1) - v(2); |
| 4 | dc(4) = -v(2); |
| 5 | dc(5) = v(2) - v(3); |
| 6 | dc(6) = -v(3); |
| 7 | dc(7) = v(3) - v(4); |
| 8 | dc(8) = -v(4); |
| 9 | dc(9) = v(4) - v(5) - v(6); |
| 10 | dc(10) = -v(5) - v(28); |
| 11 | dc(11) = v(5) + v(6); |
| 12 | dc(12) = -v(6) - v(7) - v(8) - v(27); |
| 13 | dc(13) = -v(7) - v(26); |
| 14 | dc(14) = v(7) + v(8); |
| 15 | dc(15) = -v(8) - v(9) - v(12) - v(19) + v(20) - v(25); |
| 16 | dc(16) = -v(9) + v(11) + v(16) - v(21); |
| 17 | dc(17) = v(9) + v(10) + v(19); |
| 18 | dc(18) = -v(10) + v(12) - v(20); |
| 19 | dc(19) = -v(10) - v(11) - v(13) - v(19) + v(21); |
| 20 | dc(20) = -v(11) - v(12) - v(14) + v(18) - v(24); |
| 21 | dc(21) = v(13) - v(15); |
| 22 | dc(22) = v(14) - v(15); |
| 23 | dc(23) = v(22) + v(17) - v(23); |
| 24 | dc(24) = v(15) - v(16); |
| 25 | dc(25) = -v(25) - v(26); |
| 26 | dc(26) = v(25) + v(26); |
| 27 | dc(27) = -v(27) - v(28); |
| 28 | dc(28) = v(27) + v(28); |

### References

1. Cho KH, Shin SY, Lee HW, Wolkenhauer O: **Investigations into the analysis and modeling of the TNF-α-mediated NFkappaB signaling pathway**. *Genome Res* 2003, 13:2413-2422.
2. Cho KH, Shin SY, Kolch W, Wolkenhauer O: **Experimental design in systems biology, based on parameter sensitivity analysis using a Monte Carlo method: A case study for the TNF-α-mediated NF-kappa B signal transduction pathway**. *Simulation-Transactions of the Society for Modeling and Simulation International* 2003, 79:726-739.
3. Park SG, Lee T, Kang HY, Park K, Cho KH, Jung G: **The influence of the signal dynamics of activated form of IKK on NFkappaB and anti-apoptotic gene expressions: a systems biology approach**. *FEBS Letter* 2006, 580:822-830.
