# Supplementary material for: A formal model for analyzing drug combination effects and its application in TNF-α-induced NFκB pathway
Source: BMC Syst Biol. 2010 Apr 25;4:50. doi: 10.1186/1752-0509-4-50 (PMC2873319; doi:10.1186/1752-0509-4-50)
Supplement: Additional file 4 — Proof of influence of feedback. Proof of Lemma 2 for influence of feedback structure to original serial system based on comparison principle that is used to compute bounds on solutions of differential equations. [file 1752-0509-4-50-S4.DOC]

## Additional file 4 – Summary of algorithm for TNF-α-induced NFκB pathway

***Step 1: Define the target reactions set according to literatures.***

In TNF-α-induced NFκB pathway, we choose three key nodes including Proteasome Inhibitor II Aldehyde, HSP90 inhibitor Geldanamycin and IKK-β inhibitor PS-1145. According to their inhibition mechanisms, the corresponding target reactions were determined. These consist of the target reactions set. Additional Table 5 showed the corresponding target reactions and reaction No.s in Additional Table 1.

### Table S5 Target reactions

| **Drugs** | **Targets reactions** | **Target reactions No.** |
| --- | --- | --- |
| Geldanamycin | RIP1 binding with TNFR1 complex | 3 |
| PS-1145 | IKK-β activating IκB | 9, 12 |
| Aldehyde | IκB degradation | 19, 20, 21, 24 |

***Step 2: Analyze the system structure and define the simplified system according to the positions of target reactions.***

TNF-α-induced NFκB pathway has a serial structure with feedback. Target reactions of RIP1 and IKK-β locate on the serial path and target reactions of IκB degradation locate on feedback path. Especially key node IKK-β is on the joint of serial path and feedback path (Figure 6B). Then from Lemma 1, we gained the corresponding simplification for each target to help simplifying the system as the dashed box area in Figure 6A. Table 1 showed the target reactions in simplified system.

***Step 3: Calculate the system assessment factors (combination index) for each combination on simplified system.***

Since we had the simplified system and corresponding target reactions, we could calculate the synergism assessment factors of the drug combinations based on simulation. We chose changing ratio of reaction velocity constants to reflect the inhibition effects of different dose on targets. The smaller the changing ratio is, the stronger the inhibition effect is, and that is, the dose is larger. In the simulation, we set the changing ratio ranging from 0.9 to 0.0001 fold to cover a wide dose range. Meanwhile, we set a same changing ratio for each target in the drug combination for convenience.

We ran the simulation for 100 times (i.e. 100 changing ratios), and we got 100 system assessment factors for each drug combination. We used the mean of these 100 system assessment factors to show average combination effect of each combination on a wide dose range. The results were shown in Table 2, column ' Synergism Assessment Factor - simplified system '.

***Step 4: Calculate the parameter sensitivity of forward path.***

In TNF-α-induced NFκB pathway, in order to apply Lemma1, the parameter sensitivity of IKKKβ-P concentration (since IKKKβ-P is intermediate product of process starting from RIP1 on the forward path) to changing ratio of reaction velocity constant of RIP1 binding with TNFR1 complex on the forward path should be calculated. That is the $\frac{\partial x}{\partial a}$ in Lemma 1. According to Corollary 1, the sign of this parameter sensitivity of serial structure should be negative. However, in our simulation parameter *a* is the changing ratio of reaction velocity constant, not the Michaelis-Menten kinetic constant *K_m_*. Actually, *a* is in inverse proportion to *K_m_* for competitive inhibition (based on the deduction of Michaelis-Menten kinetics). For an enzymatic reaction as follows,

$$E+S\leftrightarrow_{k_{2}}^{k_{1}} ES\to^{k_{3}} P+E$$

(*E*: Enzyme, *S*: Substrate, *P*: Product)

The Michaelis-Menten kinetic constant $K_{m}=\frac{k_{2}+k_{3}}{k_{1}}$. The competitive inhibition effect on the reaction velocity of *E* binding with *S* can be represented as the changing on the reaction velocity constant *k*_1_. In our algorithm, we took *a* as the changing ratio of *k*_1_. The Michaelis-Menten kinetic constant after inhibition $K'_{m}=\frac{k_{2}+k_{3}}{a\cdot k_{1}}=\frac{K_{m}}{a}$. From this equation we can conclude that *a* is in inverse proportion to *K_m_*. So in our simulation, the sign of this parameter sensitivity is positive. We ran simulation for 100 times, and the mean of the parameter sensitivities is 10.921.

According to Eq. 3, all these three combinations could generate synergistic effect in simplified system since the system assessment factors are negative. Since the parameter sensitivity is positive, with Eq. 6 and Lemma 1, we could conclude that the signs of corresponding synergism assessment factors of original system are also negative which means that the tree drug combinations can generate synergistic effect on the original system.

***Step 5: Calculate the system assessment factors (combination index) for each combination on original system.***

Like what we have done in Step 3, we applied the simulation on original system and got the system assessment factors shown in Table 2, column ' Synergism Assessment Factor - original system'. All the three synergism assessment factors are negative. It indicates that the three drug combinations can generate synergistic effect in the original system. These results are coincident with the conclusion on simplified system. It should be noted that in the simulations we applied the same changing ratio ranges (0.9~0.001 fold) in both simplified system and original system. However, since there is parameter sensitivity from original system to simplified system (Lemma1), the actual changing ratio range of the simplified system corresponding to that of the original system is not the same. For example, we changed (*a*, *b*) -- the parameter of original system -- to (0.9*a*, 0.9*b*) in the original system, but in the simplified system, the corresponding parameter (*x*, *y*) may not be changed to (0.9*x*, 0.9*y*). However, in the simulation, we changed (*x*, *y*) to (0.9*x*, 0.9*y*) for convenience. This difference may cause that the values of synergism assessment factors of simplified system are not the same as those of original system. However, we still could use the results of simplified system to conclude the combination effect on the original system. In the future, we will extend the simulation algorithm with the exact parameter sensitivity information that can help to define the right parameter ranges of simplified system.
